# Hydrogen Impurity Defects in Rutile TiO_2_

**DOI:** 10.1038/srep17634

**Published:** 2015-12-02

**Authors:** Li-Bin Mo, Yu Wang, Yang Bai, Qing-Yun Xiang, Qun Li, Wen-Qing Yao, Jia-Ou Wang, Kurash Ibrahim, Huan-Hua Wang, Cai-Hua Wan, Jiang-Li Cao

**Affiliations:** 1 Institute of Advanced Materials and Technology, Beijing Key Lab for Corrosion, Erosion and Surface Technology, University of Science and Technology Beijing, Beijing 100083, China; 2 Department of Applied Physics, Hong Kong Polytechnic University, Hong Kong, China; 3 Department of Chemistry, Tsinghua University, Beijing 100084, China; 4 Institute of High Energy Physics, Chinese Academy of Sciences, Beijing 100049, China; 5 Institute of Physics, Chinese Academy of Sciences, Beijing 100190, China

## Abstract

Hydrogen-related defects play crucial roles in determining physical properties of their host oxides. In this work, we report our systematic experimental and theoretical (based on density functional theory) studies of the defect states formed in hydrogenated-rutile TiO_2_ in gaseous H_2_ and atomic H. In gas-hydrogenated TiO_2_, the incorporated hydrogen tends to occupy the oxygen vacancy site and negatively charged. The incorporated hydrogen takes the interstitial position in atom-hydrogenated TiO_2_, forming a weak O-H bond with the closest oxygen ion, and becomes positive. Both states of hydrogen affect the electronic structure of TiO_2_ mainly through changes of Ti 3d and O 2p states instead of the direct contributions of hydrogen. The resulted electronic structures of the hydrogenated TiO_2_ are manifested in modifications of the electrical and optical properties that will be useful for the design of new materials capable for green energy economy.

Hydrogen has rather complex chemical states in nature including H^+^, H^0^ and H^−^, which makes its behaviors in the host materials complicated. A fuller understanding of the interactions between oxide materials and hydrogen is crucial for the research and successful applications of such materials in a broad range of scientific and technological fields from green energy techniques to electronics industries. For example, hydrogen ions are positively charged in ZnO, contributing to high conductivity^1^,^2^. In BaTiO_3_, instead, hydrogen ions are negatively charged, occupying the position of oxygen vacancies^3^. As-doped BaTiO_3-x_H_x_ is also electronically conductive and stable in air and water at ambient conditions^3^. It is also proposed that new materials or devices with novel functionalities may be designed through appropriate hydrogen treatment. All these areas are calling for a thorough understanding of the hydrogen behaviors in oxide materials and responses of the hosts to different hydrogen atmospheres.

TiO_2_ shows promising application prospects in some rapidly growing hydrogen-related technologies such as hydrogen production^4^,^5^, and solar cells^6^, etc^7^,^8^,^9^ because of its excellent properties and ready accessibility. For example, Chen *et al.*^10^ reported that hydrogenated anatase TiO_2_ nanocrystals exhibited significantly improved solar photocatalytic performances, which triggered much research interest in the hydrogen defect engineering of oxide materials. Furthermore, hydrogenated TiO_2_ nanowire arrays also possess excellent photoelectrochemical-water-splitting performance^11^ and super capacitive properties^12^. Interestingly, besides of high-temperature annealing in gaseous H_2_, treatment by atomic H at room temperatures could also significantly change performances of TiO_2_. Chester^13^ first found that the conductivity of TiO_2_ increased upon atom-hydrogenation through water electrolysis. Chen^14^ also observed similar effects in TiO_2_ and other oxides. But the proposed mechanisms for the above behaviors diverge in different researches. Chen^10^,^15^ and Naldoni^16^ regarded surface disorders induced narrowing of bandgap of TiO_2_ and therefore the enhancement of its absorption ratio in visible light range. Deford^17^, Herklotz^18^ and Kilici^19^ assumed that the interstitial hydrogen was formed in gas-hydrogenated TiO_2_ and correlated the interstitial hydrogen with the increase in conductivity and light absorption. Besides of the viewpoint that interstitial hydrogen atom was the origin of enhanced conductivity in atom-hydrogenated TiO_2_, Chester^13^ and Chen^14^ also regarded that V_O_ formed in oxide materials in hydrogen atmospheres^20^,^21^,^22^,^23^. Wang^11^ and Zhai^24^, however, regarded that V_O_ was the main reason responsible for the reduction of bandgap. Even more complex, Lu *et al.*^12^ thought that the surface hydroxyl groups of hydrogenated TiO_2_ should be responsible for the change of performances. Filippone *et al.*^25^ reported that H behaved as a deep donor in rutile and formed stable H-oxygen vacancies (V_O_) complexes. Though the defect engineering by hydrogen treatment has attracted much attention over recent years, the defect states and their atomic- and electronic structure in hydrogenated TiO_2_ are still not clearly understood so far. The above debate is partially induced, we think, by different sample structures (nano-particles, nanowires or bulk materials) and/or by different treatment conditions (high temperature annealing or room temperature electrochemical method) among different research groups. In this research, we focus only on bulk materials with single crystalline to first minimize the influence of surface disorder. Further, we applied the above two methods to treat the same batch of samples to minimize influence of diversity among different samples. The above controlled conditions will make our data more comparative and suggestive to dissolve some basic questions in this field Therefore, the present study aims at exploring the hydrogen impurity-related defect states in bulk rutile TiO_2_. Oxygen vacancies and H-oxygen vacancies (V_O_) complexes as main defects in gas-hydrogenated TiO_2_ were introduced through high-temperature annealing treatment and interstitial hydrogen as main defects in atom-hydrogenated TiO_2_ were introduced by water electrolysis. Through comparison experiments with bulk TiO_2_ samples with different defects as well as density functional theory (DFT) calculations, we hope to open up new opportunities in green energy techniques, including water splitting, dye-sensitized solar cells, and photocatalysis etc.

Figure 1 (a) shows hydrogen contents and room temperature (RT) resistivity of gas-hydrogenated rutile TiO_2._ The samples treated with P_H2_ = 0 were named as vacuum-annealed samples. Their resistivity decreases by more than seven orders of magnitude to several Ω cm, which could be attributed to the formation of V_O_^22^,^23^. With increasing P_H2_ the incorporated hydrogen content increases and resistivity decreases monotonically. Hall measurements show that the carriers in these high temperature-annealed samples are all electrons (Supplemental Fig. S1), demonstrating formation of donor defects. Carrier mobility in the 0.95 bar gas-hydrogenated sample is 42.3 cm^2^ V^−1^ s^−1^ at 30 K, lower than 94.5 cm^2^ V^−1^ s^−1^ of the vacuum-annealed sample. Fig. 1(b) plots the temperature-dependence of the resistivity between 2 K and 300 K. Different from the blank sample, the resistivity of the high temperature-annealed samples first decreases with decreasing temperature and then increases sharply after reaching a minimum around 30 K. Here, the temperature dependence of the resistivity above 30 K is proposed to be determined mainly by the carrier mobility. Below 30 K, the donors start to be frozen out from conduction band (CB). The resistivity exhibits an exponential dependence on 1/T, following the equation ρ = ρ_0_exp(–ΔE_D_/k_B_T)^26^. And fittings show that the ionization energy of defects ΔE_D_ of all high temperature-annealed samples are 6.5 ± 1.5 meV. It indicates that their dominant defects could be the same, i.e. V_O_. Similarly, Yagi^23^ *et al.* reported a ΔE_D_ of ~5 meV for V_O_ in rutile TiO_2_. The resistivity of the samples annealed in 0.35 and 0.95 bar H_2_ at high temperature increases with further decreasing temperature, following Mott’s law ρ = ρ_1_exp(T_0_/T)^1/4^. Their conductivity mechanism could be attributed to the variable range hopping conduction^26^,^27^. The higher P_H2_ leads to a higher donor density and finally produces a defect band in the band gap. Consequently, electrons could hop between donors through thermal-assisted tunneling, which answers for the residual conductivity below 4 K in the higher P_H2_-hydrogenated TiO_2_.

TiO_2_ single crystals with interstitial H (H_i_) defects fabricated by atom hydrogenation at RT show a much reduced RT resistivity of 14.5 Ω cm and a similar temperature-dependence with the high temperature-annealed samples (Fig. 1b). The ΔE_D_ of atom-hydrogenated TiO_2_ has not been reported before. Here, a shallow donor level with a ΔE_D_ of 3.9 meV is measured, smaller than that in the high temperature-annealed samples. Thus, it is inferred that the defects in atom-hydrogenated TiO_2_ are different from those in gas-hydrogenated TiO_2_. Furthermore, carrier mobility of the atom-hydrogenated sample is 7.71 cm^2^ V^−1^ s^−1^ (Supplemental Fig. S1), much smaller than that of vacuum-annealed sample and that of gas-hydrogenated TiO_2_, which further indicates different scattering defects in samples treated by the different methods.

Figure 2a shows the Fourier transform infrared (FTIR) absorption spectra. The blank sample has strong absorption between 400 ~ 1300 cm^−1^ as normal^28^,^29^. In contrast, the transparency of hydrogenated samples increases significantly. Interestingly, the 0.95 bar gas-hydrogenated sample becomes almost completely infrared transparent in the full range of measurement. Note that the gas-hydrogenated TiO_2_ also has high conductivity and could be potentially used as transparent conductors in the infrared band. A strong absorption peak at 3280 cm^−1^ is observable for the atom-hydrogenated sample. This peak should be the stretch mode of O-H bonds in rutile TiO_2_^18^,^28^. The O-H absorption peak in atom-hydrogenated TiO_2_ disappears after a 10 min-dehydrogenation in air at 700 ºC, as shown in the inset of Fig. 2a. Furthermore, high resistivity of the atom-hydrogenated TiO_2_ recovered gradually to the virgin state after a few days aging at RT, similar with previous report^30^, whereas little changes can be observed in the gas-hydrogenated TiO_2_ after a five-day aging. Therefore, the incorporated hydrogen in atom-hydrogenated TiO_2_ is more diffusible and unstable than that in gas-hydrogenated TiO_2_. By contrast, no such an absorption peak can be observed for the gas-hydrogenated samples.

Figure 2b presents optical absorption spectra of the samples. Upon hydrogenation or vacuum-annealing, the absorption edge shifts to the lower energy side and the absorption ratio for photons with lower energy than the absorption edge increases significantly. Meanwhile, colors of vacuum-annealed samples and the atom-hydrogenated TiO_2_, turn from light yellow of blank sample to light blue (Fig. 2c) and the color of gas-hydrogenated sample becomes even dark blue, similar with the case of gas-hydrogenated anatase TiO_2_ nanocrystals^10^. The valence-band (VB) spectra were measured by synchrotron radiation x-ray photoelectron spectroscopy (XPS) (Fig. 2d). For the vacuum-annealed and hydrogenated samples, the VB maximum lies at 2.6 eV and 1.4 eV, respectively, which are closer to the Femi level than blank sample as reported by Chen^10^. The results suggest a narrowing of the band gap, in agreement with the optical absorption measurements in Fig. 2b. Naldoni^16^ and Zuo^31^ regarded that V_O_ contributed energy levels 0.7 ~ 1.0 eV below conduction band. Chen^10^ and Naldoni^16^ regarded that surface disorder contributed levels 2.0 eV below conduction band. In our VB spectra, we also observed a band 1.4 eV below Fermi level in samples annealed in 0.95 bar H_2_ atmospheres. Instead, the VB position of the atom-hydrogenated TiO_2_ was 2.5 eV. And the VB position of vacuum-annealed samples was also about 2.6 eV. These results made us propose that different hydrogen defects formed in different treatment methods. Furthermore, O-H bonds only formed in atom-hydrogenated TiO_2_ but not in gas-hydrogenated samples. Instead, a large amount of V_O_ and H defects coexisted in the gas-hydrogenated samples. Therefore we propose oxygen vacancies and hydrogen ions coexist independently or correlatedly in the gas-hydrogenated samples while only interstitial hydrogen atoms exist in the samples treated by electrochemical method. In order to understand different existing forms of hydrogen defects, we have turned to DFT calculations.

DFT calculations were carried out using the Vienna ab initio Simulation Program (VASP) code with projector-augmented-wave (PAW) pseudopotentials and the Perdew-Burke-Ernzerhof (PBE) exchange-correlation functional. The rutile TiO_2_ structure was described as a 3 × 3 × 3 supercell. First, the density of states (DOS) and the partial density of states (PDOS) of a perfect TiO_2_ supercell (Fig. 3a) and the supercell with V_O_ were calculated (Fig. 3b). As compared to the perfect TiO_2_, the introduction of V_O_ in the supercell results in two loosely captured electrons by three Ti dangling bonds. These two electrons can be transferred to Ti 3d states^32^. Then a H atom was incorporated in the supercell with V_O_ and perfect supercell, respectively. It was found after structure optimization that the H atom added in the supercell with V_O_ tends to reside on the V_O_’s site as H_O_ and the resulting lattice distortion becomes smaller than that with V_O_ alone. This finding agrees with our synchrotron radiation x-ray diffraction results (Supplemental Fig. S3). The Fermi level for TiO_2_ with H_O_ also lies in the conduction band (CB) owing to the contribution of Ti 3d electrons (Fig. 3c). The inset shows that the states of H_O_ contribute mainly to the VB. By contrast, H in the perfect TiO_2_ prefers the interstitial site of oxygen octahedral as H_i_, which is consistent with an earlier report^33^. The distance from H_i_ to the closest O is found to be 0.991 Å, very similar to the bond length of hydroxyl in H_2_O. H_i_ can also affect the Ti 3d band structure and move the Fermi level into the CB. The inset of Fig. 3d shows that some states of H_i_ appear in the CB. Note that DOS of interstitial H is very small and only as 1% as that of H defects around Oxygen vacancies. In all these configurations, the VB and the CB are still primarily formed by O 2p and empty Ti 3d states, respectively. Hydrogen defects have little direct influence on the structure of VB or CB, contributing few DOS near the bandgap. However, they could modulate the structure of TiO_2_ indirectly by their hybridization with O 2p and Ti 3d orbits. Chen^10^ and Naldoni^16^ proposed this shift was due to surface disorders. Here we tend to ascribe this shift in bulk and single crystalline TiO_2_ to hybridization of H with O 2p orbits. This result agrees with our previous work^34^.

The charge density and differential charge density analyses show that electrons move from V_O_ to the neighboring Ti and O atoms in TiO_2_ with V_O_ (Fig. 4a,d). When H stays on the site of V_O_, the neighboring Ti and O atoms give electrons to H_O_, making H_O_ negatively charged (Fig. 4b,e). Bader analyses also show that H_O_ has 1.50 valence electrons (supplemental Table 1). In TiO_2_ with H_i_, the charge density of H_i_ and the closest O atoms overlaps (Fig. 4c) and the charge density in between them is high (Fig. 4f), revealing the formation of a covalent bond, or specifically an O-H bond. The valence electron of H_i_ is 0.35 and similar with that of H atoms in H_2_O. These DFT calculations coincide well with our experimental observations of the appearance of the 3280 cm^−1^ IR absorption peak in atom-hydrogenated TiO_2_, but not in gas-hydrogenated TiO_2_. Higher hydrogen contents in the supercell do not bring about significant charge density differences. Indeed, no experimental and theoretical evidences were found for the existence of O-H bonds in gas-hydrogenated TiO_2_.

Annealing in vacuum and gaseous H_2_ can both introduce a shallow donor state in rutile TiO_2_ with nearly the same ionization energy of 6.5 ± 1.5 meV. The H_i_ alone in atom-hydrogenated TiO_2_ also introduces a shallow donor, however, with lower ionization energy of 3.9 meV. Note that the H_i_ is weakly bonded to the closest O atom and the resulted O-H bonds could be easily broken by high temperature annealing in air in short time or by several days’ aging at RT^14^. This hydrogenation-derived O-H bonds behave quite differently from those inherent O-H defects in earlier studies in flame fusion-derived rutile TiO_2_ crystals^28^,^29^ or natural ores^35^ which contained unavoidably large contents of OH defects, or the deuteration was carried out in D_2_O through isotope exchange^36^. Those inherent hydrogen defects were rather stable against high temperature annealing^26^. The H_O_ defects, or the combination of H atoms with V_O_ could stabilize the hydrogen atoms in TiO_2_, making it insensitive to RT aging. Our study offers useful insights for studies of the interplay of hydrogen atmospheres and solid state oxide materials. We expect this research could contribute to the property tailoring of TiO_2_ through appropriate hydrogen-related defect engineering.

## Methods

Rutile TiO_2_ single crystals of (001)-orientation synthesized by floating zone method were used. All the samples were annealed in air at 700 °C for 120 hours to eliminate the native defects and reached a RT equilibrium resistivity of (2.0 ± 0.1) × 10^8^ Ω cm. For high-temperature annealing, the samples were sealed in pre-evacuated quartz ampoules (background pressure <1.0 × 10^−9^ bar) with 99.999% H_2_ of a RT pressure of 0, 0.05, 0.35 and 0.95 bar, respectively. Then the samples were annealed at 600 °C for 50 hours to reach their equilibrium state, followed by air quenching to RT. It is worth mentioning that the infrared spectra would be significantly affected by the hydroxyl pollution from quartz ampoules. The FTIR spectra obtained in the samples annealed in vacuum in normal quartz tubes also showed an observable absorption peak at 3280 cm^−1^ (as shown in Supplemental Fig. S3). In order to avoid possible hydroxyl pollution from quartz ampoules, we have chosen dehydroxylated quartz ampoules. Furthermore, flame fusion-derived rutile TiO_2_ single crystals unavoidably contained large contents of OH defects^28^,^29^, so we chose the rutile single crystals synthesized by floating zone method. Moreover, high pressure hydrogen gas can also help to avoid hydroxyl pollution from quartz ampoules.

For comparison, interstitial hydrogen was introduced by atom-hydrogenation through electrolysis of 0.1 M Na_2_SO_4_ water solution at RT^22^. The TiO_2_ chips were electroded by firing Ag pastes on parts of the chips. Atomic H was generated on the Ag electrode and then diffused into TiO_2_ during the hydrogenation. The TiO_2_ crystals became gradually blue and hydrogen started to form directly on the TiO_2_ crystals. A surface layer of 50 μm thick was removed in order to keep the surface clean for following analyses.

The resistivity and Hall Effect of the samples were measured from 2 to 300 K by Physical Property Measurement System 9 (PPMS-9) and the current–voltage (*I*–*V*) characteristics were measured by Keithley 2400 using a four-electrode method. The oxygen loss was calculated through weight measurement using a micro-balance. The crystal structures were examined by synchrotron radiation x-ray diffraction (XRD) at 1W1A of Beijing Synchrotron Radiation Facility (BSRF). The optical absorption spectra of rutile TiO_2_ samples were measured by Fourier-transform infrared spectrometer (Nicolet 6700) under transmission mode and Double beam UV-visible spectrophotometer (TU-1901) under reflection mode. The valence band structures were examined by synchrotron radiation XPS at 4B9B of BSRF. Hydrogen contents of samples were determined by diffusible hydrogen analyzer (Bruker-G4 Phoenix) at 700 °C.

The electronic structures of TiO_2_ with different defects were calculated based on density functional theory (DFT) with the Perdew–Burke–Ernzerhof (PBE) exchange-correlation functional using the Vienna *ab initio* Simulation Program (VASP) code with projector-augmented-wave (PAW) pseudopotentials. The rutile TiO_2_ structure was described as a 3 × 3 × 3 supercell with 162 atoms consisting of a twelve-trilayer slab. The cutoff energy for expanding the Kohn-Sham wave functions was 400 eV. We used the experimentally derived lattice constants (a = b = 4.595 Å, c = 2.959 Å). All the atoms were fully relaxed without constraints until the forces were converged to 0.01 eV/Å. The Monkhorst-Pack *k*-point grid was 5 × 5 × 9 for Brillouin zone integration of the supercell which was tested to be well converged.

## Additional Information

**How to cite this article**: Mo, L.-B. *et al.* Hydrogen Impurity Defects in Rutile TiO_2_. *Sci. Rep.* **5**, 17634; doi: 10.1038/srep17634 (2015).

## Supplementary Material

Supplementary Information

## Figures and Tables

**Figure 1:**
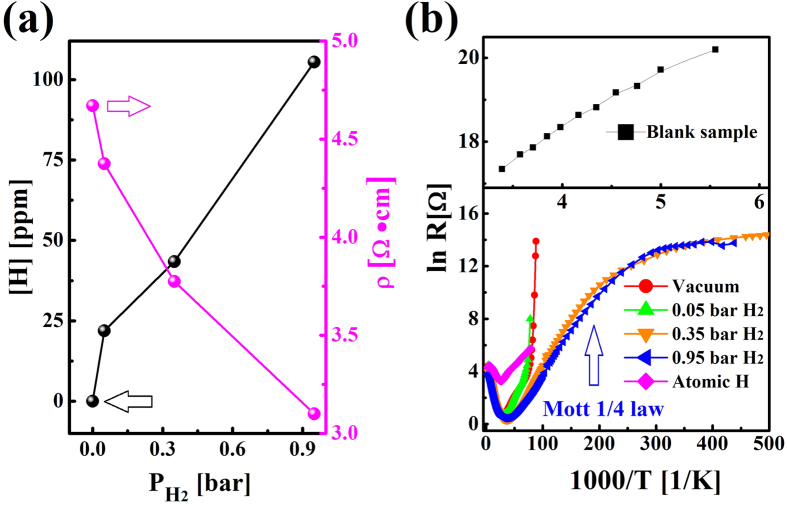
Molar content of incorporated hydrogen and transport properties of hydrogenated TiO_2_ single crystals. (**a**) Changes of the hydrogen content and RT resistivity of TiO_2_ with P_H2_ upon gas-hydrogenation. An oxygen loss of about 300 ppm is measured for the 0.95 bar gas-hydrogenated rutile TiO_2_, which is approximately 3 times of the hydrogen content. (**b**) temperature-dependences of resistivity from 2 K to 300 K.

**Figure 2:**
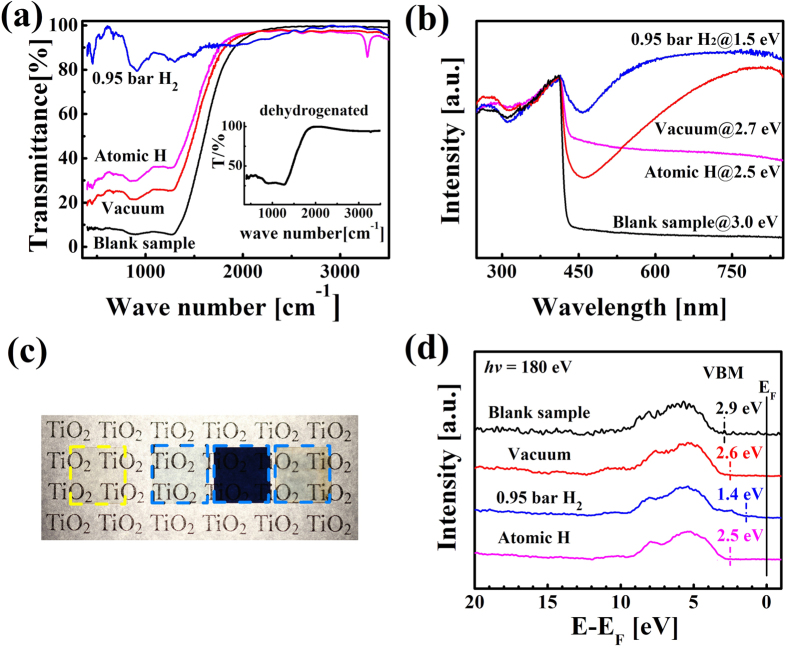
Characterizations of TiO_2_ single crystals. (**a**) FTIR absorption spectra. The inset shows the FTIR spectrum of the atom-hydrogenated TiO_2_ after a 10 min-dehydrogenation at 700 ºC. (**b**) Optical absorption spectra. The band gap values are given. (**c**) Optical images of the blank, vacuum-annealed, gas-hydrogenated and atom-hydrogenated samples (from left to right). (**d**) Valence-band spectra of synchrotron radiation.

**Figure 3:**
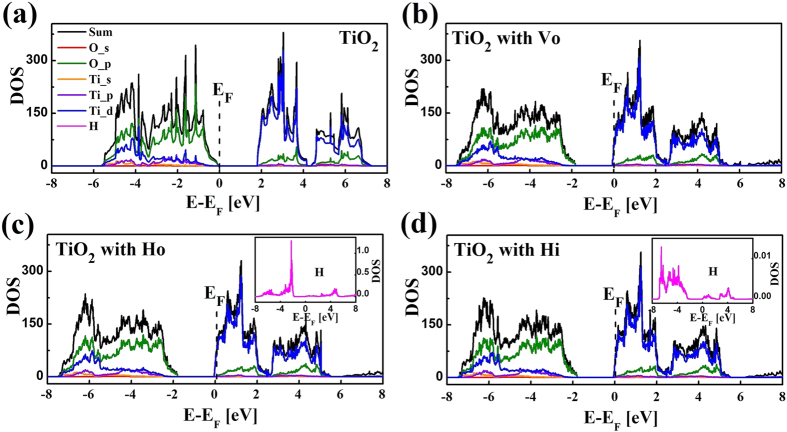
DFT calculations. The DOS and PDOS of (**a**) the perfect rutile TiO_2_ and (**b**) TiO_2_ with V_O_ in simulation of vacuum-annealed TiO_2_. In our calculations, the intrinsic band gap of rutile TiO_2_ is 1.7 eV, in agreement with reported values^37^,^38^. (**c**) Gas-hydrogenated TiO_2_ with H staying at V_O_ which corresponds to the minimum energy state. (**d**) Atom-hydrogenated TiO_2_ with H_i_. The insets of (**c**) and (**d**) show the PDOS of the hydrogen, respectively.

**Figure 4:**
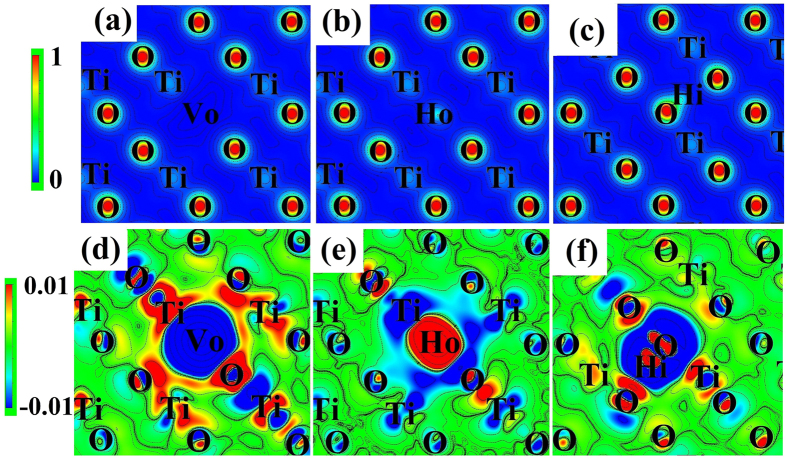
Charge density and differential charge density for different configurations. (**a**–**c**) is the charge density of the perfect TiO_2_, TiO_2_ with V_O_, TiO_2_ with H_O_, and TiO_2_ with H_i_, respectively. (**d**–**f**) is the differential charge density of TiO_2_ with V_O_, TiO_2_ with H_O_, and TiO_2_ with H_i_, respectively. The blue color indicates the low electron density and the red color indicates the high electron density.
