# Supplementary material for: Hydrogen Impurity Defects in Rutile TiO2
Source: Sci Rep. 2015 Dec 2;5:17634. doi: 10.1038/srep17634 (PMC4667266; doi:10.1038/srep17634)
Supplement: Supplementary Information [file srep17634-s1.pdf]

Supplemental Information

Hydrogen Impurity Defects in Rutile TiO_2

Li-Bin Mo,[1] Yu Wang,[2] Yang Bai,[1] Qing-Yun Xiang,[1] Qun Li,[1] Wen-Qing Yao,[3] Jia-Ou Wang,[4] Kurash Ibrahim,[4] Huan-Hua Wang,[4] Cai-Hua Wan[5] and Jiang-Li Cao[1,*]

[1]Institute of Advanced Materials and Technology, University of Science and Technology Beijing, Beijing 100083, China

[2]Department of Applied Physics, Hong Kong Polytechnic University, Hong Kong, China

[3]Department of Chemistry, Tsinghua University, Beijing 100084, China

[4]Institute of High Energy Physics, Chinese Academy of Sciences, Beijing 100049, China

[5]Institute of Physics, Chinese Academy of Sciences, Beijing 100190, China

*Author to whom correspondence should be addressed. Email: perov@sina.com

Fig. S1 Hall effect measurements of rutile TiO_2 single crystals at low temperatures. (a) Vacuum-annealed TiO_2, (b) gas-hydrogenated TiO_2, and (c) atom-hydrogenated TiO_2. The carrier concentration n, the resistivity ρ and the carrier mobility μ were obtained through linear fitting between the measured Hall resistance and the applied magnetic field.

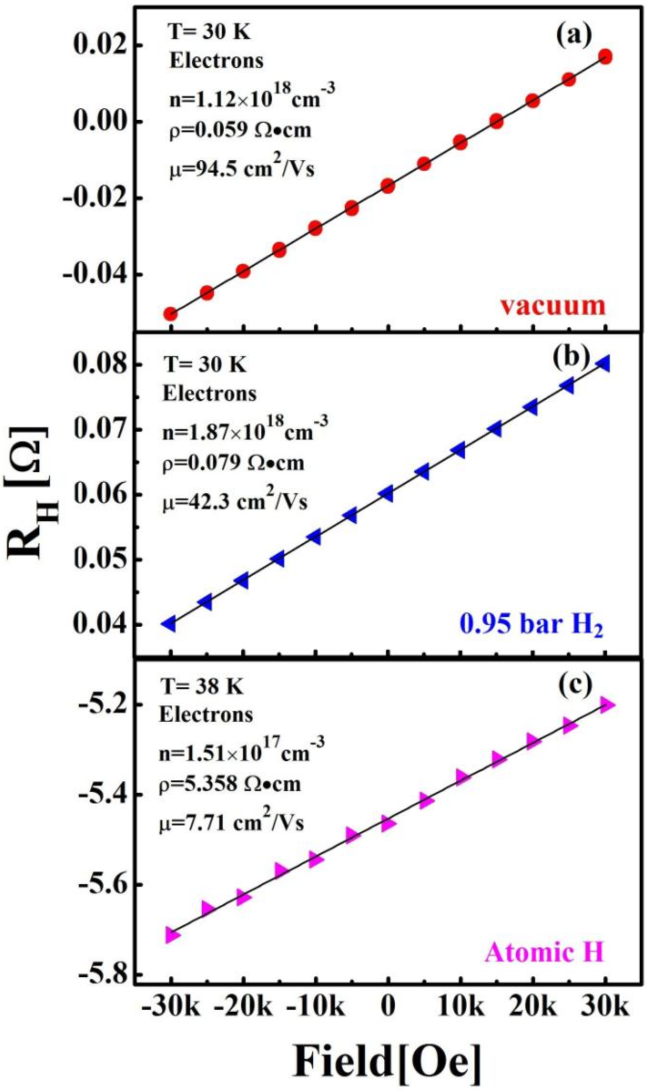

Fig. S2 XRD patterns of rutile TiO_2 samples. The TiO_2 single crystals were ground into powders for the XRD measurements.

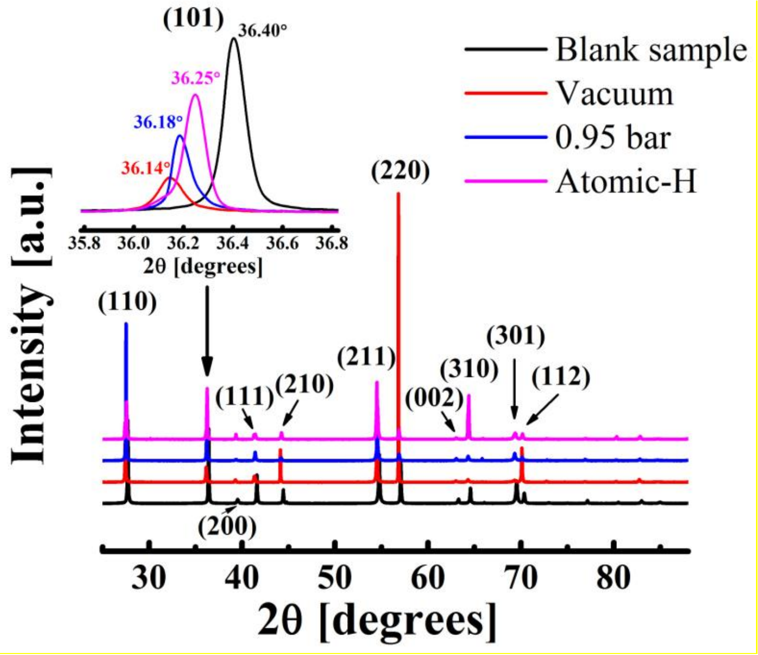

Fig. S3 Infrared absorption spectra of annealed TiO_2.

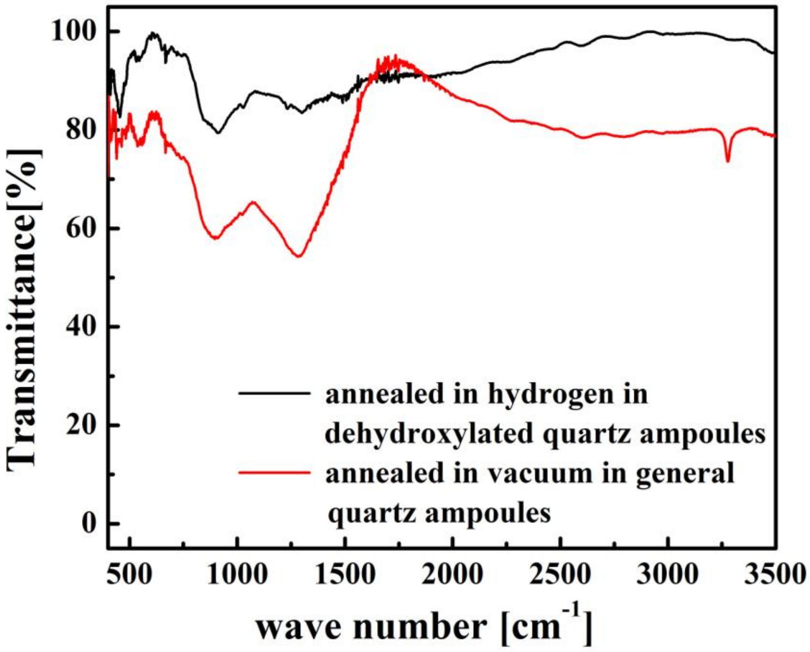

Table I Bader analyses.

Atom	Bader charge	Distance	Atom	Bader charge	Distance
**Blank sample**			O_4	7.0288	0.8484
O	6.9767	0.844	Ti_1	2.0386	0.8313
Ti	2.0453	0.886	Ti_2	2.0316	0.8761
**Vo**			Ti_3	2.0390	0.8326
O_1	7.0156	0.8447	Ti_4	2.0317	0.8705
O_2	7.0156	0.8774	**Ho**		
O_3	7.0156	0.8405	H	1.5039	0.7438
Ti_1	2.0953	0.8041	O_1	6.9946	0.8447
Ti_2	2.0953	0.8041	O_2	6.9946	0.8447
Ti_3	2.0987	0.8128	O_3	6.9874	0.8412
**Hi**			O_4	6.9875	0.8412
H	0.3565	0.0704	Ti_1	2.0788	0.8322
O_1	6.9981	0.8248	Ti_2	2.0787	0.8312
O_2	7.1875	0.7024	Ti_3	2.0925	0.8601
O_3	7.0013	0.8284			
